# Adalimumab Therapy for Recalcitrant Pyoderma Gangrenosum

**Published:** 2006-11-20

**Authors:** Margaret A. Fonder, Deborah L. Cummins, Benjamin D. Ehst, Grant J. Anhalt, Jon H. Meyerle

**Affiliations:** Department of Dermatology, Johns Hopkins Medical Institutions, Baltimore, MD

## Abstract

**Objective:** To describe a patient with treatment-refractory pyoderma gangrenosum and the outcome of a novel therapeutic approach. **Methods:** Case report and review of the literature. **Results:** A patient with inflammatory bowel disease developed severe pyoderma gangrenosum while receiving treatment with the chimeric anti-TNF-α antibody infliximab. Despite subsequent trials of numerous immunosuppressive and immunomodulatory medications, the dermatologic disease progressed. The patient's ulcers finally resolved when treatment with adalimumab, a fully humanized monoclonal antibody specific for TNF-α, was initiated. **Conclusions:** We report a novel application of the TNF-α inhibitor, adalimumab, in the treatment of pyoderma gangrenosum.

Pyoderma gangrenosum (PG) is a neutrophilic dermatosis associated in 70% of cases with underlying systemic disease such as inflammatory bowel disease (IBD), rheumatoid arthritis (RA), monoclonal gammopathy, or malignancy.^1^,^2^ Treatment of the underlying disease process promotes PG resolution in many cases. Corticosteroids are considered first-line therapy for PG.^3^ Other immunosuppressive and immunomodulatory agents, including cyclosporine, thalidomide, tacrolimus, mycophenolate mofetil, and azathioprine, are used as well. Despite multiple therapeutic options, many cases of PG are refractory to treatment.

Pharmacologic inhibition of the pro-inflammatory cytokine TNF-α has demonstrated efficacy for a wide range of inflammatory conditions, including IBD, RA, and psoriasis. There are three TNF-α inhibitors commercially available: etanercept (Enbrel, Immunex Corporation, Thousand Oaks, CA), a fusion protein dimer of the human TNF-α receptor; infliximab (Remicade, Centocor Incorporated, Horsham, PA), a chimeric mouse-human monoclonal antibody to TNF-α; and adalimumab (Humira, Abbott Laboratories, Abbott Park, IL), a fully human monoclonal antibody to TNF-α. There have been multiple recent reports of PG successfully treated with infliximab^4^–^12^ and etanercept,^13^–^15^ including one randomized controlled trial of infliximab.^4^ There is also one case report of adalimumab for a patient with idiopathic PG who had previously suffered an anaphylactoid reaction to infliximab and failed etanercept therapy.^16^

Here we present the case of a patient with IBD who developed PG despite receiving treatment with infliximab. This patient subsequently failed numerous trials of various other immunosuppressive and immunomodulatory regimens. We describe our experiences with the use of adalimumab in this patient.

## REPORT OF A CASE

A 38-year-old white woman with a 2-year history of IBD developed a rapidly enlarging, painful ulcer on her anterior left thigh. For the preceding 6 months, the patient had been on azathioprine 100 mg daily and infliximab 5 mg/kg infusions once every 8 weeks for active lymphocytic ileitis. Physical examination revealed a solitary, deep ulcer on her anterior thigh that was 2.3 cm in diameter, with a characteristic violaceous undermined border and a painful zone of induration extending 1 cm beyond the ulcer rim. Tissue cultures from a biopsy of the ulcer edge were negative for bacteria, mycobacteria, and fungi. The skin biopsy showed necrosis, a mixed inflammatory infiltrate, and a small-vessel leukocytoclastic vasculitis consistent with PG. Special stains for organisms were negative.

**Figure 1:**
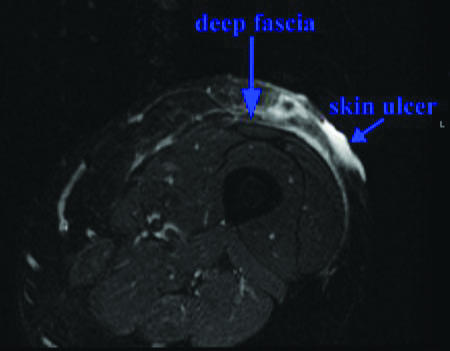
Limb MRI. STIR axial MRI of the left lower extremity demonstrating superficial T2 bright signal compatible with inflammation involving the subcutaneous tissue down to the level of the deep fascia.

The patient's PG was resistant to multiple treatment regimens. In addition to the immunosuppressive regimen for her IBD (azathioprine and infliximab), local treatments were initiated, including triamcinolone injections (5 mg/mL) to the ulcer site, topical tacrolimus 0.1% ointment twice daily, and conservative wound care including oral antibiotics. Initially, the decision was made not to administer systemic corticosteroids as the patient had previously developed pseudotumor cerebri with ocular manifestations and headache while receiving prednisone for her IBD. The PG continued to progress over the next month, so the frequency of infliximab 5 mg/kg infusions was increased to monthly administration. Monthly high-dose intravenous immunoglobulin (IVIG, 2 g/kg administered in divided doses over 3 days) was also initiated. When the ulcer depth progressed to the level of the deep fascia (Fig 1), requiring significant narcotic analgesia for pain control, cyclosporine 3 mg/kg daily was added as well.

**Figure 2:**
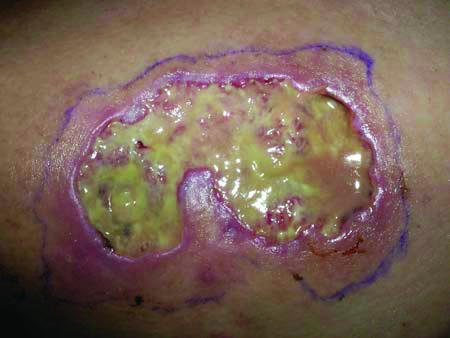
Pyoderma gangrenosum. Large purulent ulcer with violaceous undermined borders.

The regimen of cyclosporine, infliximab, azathioprine, and monthly IVIG initially resulted in improvement of the PG and some re-epithelialization; however, the immunosuppression had to be temporarily withdrawn after the patient was hospitalized for aseptic meningitis. After discharge, the IVIG was restarted, but was then stopped after 2 episodes of intractable nausea following IVIG infusions. Despite reinitiating cyclosporine and infliximab, the ulceration and surrounding inflammation progressed, eventually involving an area 8 cm in diameter on her left thigh (Fig 2). A trial of sulfasalazine 2000 mg (or 2 grams) per day was ineffective as well. Given that her PG was not responding to multiple immunosuppressive medications, prednisone 20 mg daily was initiated in consultation with ophthalmology. Higher doses of prednisone were attempted, but the patient again developed symptoms of pseudotumor cerebri. Because of the patient's persistent headaches and visual changes, the prednisone was tapered.

**Figure 3:**
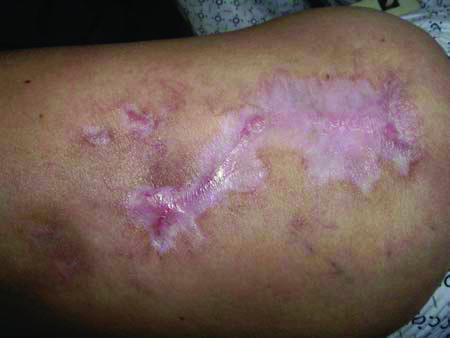
Healed wound. Residual scarring at area of pyoderma gangrenosum resolution.

While on infliximab, cyclosporine, and azathioprine, the patient required hospitalization for a flare of her IBD and intractable pain from her PG. Adalimumab was then initiated at 80 mg subcutaneous (SQ) injections every other week in combination with cyclosporine, prednisone, and sulfasalazine. The ulcer and gastrointestinal disease responded rapidly (Fig 3), and the patient was discharged on cyclosporine, prednisone, and adalimumab. Over the next 3 months, cyclosporine and prednisone were tapered and eventually discontinued as the PG continued to improve. The patient has experienced no recurrence of her PG during 4 months on adalimumab monotherapy (80 mg SQ every other week), though there have been interval flares of her bowel disease.

## DISCUSSION

Pyoderma gangrenosum is a type of painful cutaneous ulcer associated with underlying systemic disease in the majority of cases. Though the pathophysiology is poorly understood, PG is thought to be related to immune dysregulation, including defects in neutrophil chemotaxis, neutrophil hyperreactivity and overexpression of cytokines such as interleukin-8.^17^,^18^ Many of these effects may be mediated by the proinflammatory cytokine TNF-α.

TNF-α has been shown to enhance neutrophil activation, upregulate the expression of adhesion molecules, and induce the release of chemokines and cytokines from fibroblasts.^13^,^19^ The TNF-α inhibitors infliximab, etanercept, and adalimumab reduce inflammation by binding and inactivating free TNF-α.^20^ Infliximab and adalimumab have the additional ability to bind surface-bound TNF-α, with subsequent complement fixation and cell lysis, potentially suppressing T cells and other cells expressing surface TNF.^21^

Here we describe a patient who developed severe PG despite receiving treatment with infliximab and azathioprine, whose skin disease proved refractory to multiple subsequent trials of other immunomodulatory agents as well. Ultimately, this patient's PG responded rapidly and dramatically to adalimumab therapy. This case is intriguing because of the patient's disparate responses to the 2 anti-TNF-α agents.

We at first suspected that infliximab failed to suppress PG in our patient because of its immunogenicity as a mouse-human chimeric protein and the resultant formation of human antichimeric antibodies (HACAs), as infliximab had been administered for 6 months when the lower-extremity ulceration first appeared. In studies, 10%–61% of patients receiving infliximab therapy develop HACAs, which have been associated with lower serum infliximab concentrations, decreased duration of treatment response, and decreased infliximab efficacy.^22^–^25^ In contrast, the fully human agent adalimumab has the theoretical advantage of causing less antidrug antibody formation, a notion supported by experimental assays demonstrating that only 6%–12% of patients develop anti-adalimumab antibodies.^26^,^27^ However, serum assays performed 7 months after infliximab discontinuation in this patient revealed no detectable HACAs. It is possible that HACAs once present had since disappeared, but is perhaps more likely that another, as yet uncharacterized, difference between infliximab and adalimumab contributed to their divergent effects in our patient.

Studies suggest that of the TNF-α inhibitors, infliximab may be most prone to loss of efficacy over time. Finckh et al^28^ found that in patients with RA, the problem of gradual drug failure is greater for infliximab than for etanercept or adalimumab, although this study did not assess levels of antidrug antibodies. Cases of RA and Crohn's disease that become refractory to infliximab over time often respond well to adalimumab.^26^,^29^–^32^ In fact, the level of therapeutic efficacy achieved with adalimumab in infliximab-resistant patients is comparable to the improvement achieved when adalimumab is used in TNF-α-inhibitor–naïve patients.^29^,^31^ Thus, adalimumab may be a valuable treatment for PG patients who fail to respond to infliximab and other conventional immunosuppressive regimens.
